# Enhancing Clinical Information Display to Improve Patient Encounters: Human-Centered Design and Evaluation of the Parkinson Disease-BRIDGE Platform

**DOI:** 10.2196/33967

**Published:** 2022-05-06

**Authors:** Ethan G Brown, Erica Schleimer, Ian O Bledsoe, William Rowles, Nicolette A Miller, Stephan J Sanders, Katherine P Rankin, Jill L Ostrem, Caroline M Tanner, Riley Bove

**Affiliations:** 1 University of California San Francisco Weill Institute for Neurosciences University of California San Francisco San Francisco, CA United States; 2 Department of Psychiatry University of California San Francisco San Francisco, CA United States; 3 Parkinson Disease Research, Education, and Clinical Center San Francisco Veterans Affairs Medical Center San Francisco, CA United States

**Keywords:** human-centered design, personal health record, visualization in eHealth, Parkinson disease, digital health

## Abstract

**Background:**

People with Parkinson disease (PD) have a variety of complex medical problems that require detailed review at each clinical encounter for appropriate management. Care of other complex conditions has benefited from digital health solutions that efficiently integrate disparate clinical information. Although various digital approaches have been developed for research and care in PD, no digital solution to personalize and improve communication in a clinical encounter is readily available.

**Objective:**

We intend to improve the efficacy and efficiency of clinical encounters with people with PD through the development of a platform (PD-BRIDGE) with personalized clinical information from the electronic health record (EHR) and patient-reported outcome (PRO) data.

**Methods:**

Using human-centered design (HCD) processes, we engaged clinician and patient stakeholders in developing PD-BRIDGE through three phases: an inspiration phase involving focus groups and discussions with people having PD, an ideation phase generating preliminary mock-ups for feedback, and an implementation phase testing the platform. To qualitatively evaluate the platform, movement disorders neurologists and people with PD were sent questionnaires asking about the technical validity, usability, and clinical relevance of PD-BRIDGE after their encounter.

**Results:**

The HCD process led to a platform with 4 modules. Among these, 3 modules that pulled data from the EHR include a longitudinal module showing motor ratings over time, a display module showing the most recently collected clinical rating scales, and another display module showing relevant laboratory values and diagnoses; the fourth module displays motor symptom fluctuation based on an at-home diary. In the implementation phase, PD-BRIDGE was used in 17 clinical encounters for patients cared for by 1 of 11 movement disorders neurologists. Most patients felt that PD-BRIDGE facilitated communication with their clinician (n=14, 83%) and helped them understand their disease trajectory (n=11, 65%) and their clinician’s recommendations (n=11, 65%). Neurologists felt that PD-BRIDGE improved their ability to understand the patients’ disease course (n=13, 75% of encounters), supported clinical care recommendations (n=15, 87%), and helped them communicate with their patients (n=14, 81%). In terms of improvements, neurologists noted that data in PD-BRIDGE were not exhaustive in 62% (n=11) of the encounters.

**Conclusions:**

Integrating clinically relevant information from EHR and PRO data into a visually efficient platform (PD-BRIDGE) can facilitate clinical encounters with people with PD. Developing new modules with more disparate information could improve these complex encounters even further.

## Introduction

Although numerous digital health instruments have been developed, technology has thus far underdelivered when it comes to synthesizing clinical information in a coherent and an efficient way so that a clinician can use the same at the point of care (POC). As people with Parkinson disease (PD) experience problems in many different clinical domains—including motor, autonomic, cognitive, and sleep difficulties [1]—clinicians are faced with the challenge of soliciting and managing a broad range of symptoms. Medication management in people with PD is often highly personalized and based on symptoms that can change frequently, requiring detailed discussions in the clinic. Methods to quantify and track symptoms, whether through in-home mobile apps [2] or more granular quantitative metrics [3], have gained traction in PD and will present even more data streams requiring integration at the POC. Successful incorporation of this information into the clinical workflow, integrated with the already overwhelming amount of information available in the electronic health record (EHR) [4,5], is a critical challenge to overcome if clinicians are to deliver personalized and efficient care.

Researchers have addressed similar issues for other clinical conditions through platforms and dashboards that synthesize information from the EHR. Visualization dashboards have been used to manage the multiple information streams in intensive care units, where they reduce cognitive load [6] and improve quality metric adherence [7], which is variable in neurology [8]. The time required for an inpatient neurology consultation shortened after implementation of a clinical data review platform that integrated clinical information with vital signs, imaging results, and lab findings [9]. Recently, a clinician- and patient-facing platform was designed for multiple sclerosis to serve as a personalized visual aid for understanding the disease course [10]. A framework has also been proposed for integrating questionnaires administered outside usual clinical workflow to supplement the information in the EHR [11]. However, no standard tool with these capabilities is available that is tailored to the complex issues that arise in PD.

To meet this need, we developed a dashboard (PD-BRIDGE) that could be launched directly from the EHR to facilitate clinical interactions with people with PD. Our goal is to improve the efficiency and efficacy of clinical encounters with patients with PD. These encounters are highly complex because the wide-ranging symptoms people with PD experience demand multiple types of information to be considered for management, and this substantial time is needed to solicit and document these types of information. Any tool with this focus must be designed considering the intended users (ie, clinicians or patients). Such an instrument would be most effectively developed using human-centered design (HCD). HCD, when applied to digital health technology, describes a process that starts with identifying the needs of all stakeholders involved in the system that the technology hopes to change, continues with iterative feedback, and finally accounts for how the outcomes of the digital intervention compare with the intended goals [12]. HCD seeks to reduce the reluctance and delay with which many health technologies are adopted into daily practice [13].

Therefore, we used HCD to design and develop a platform that integrates various data types from the EHR, as well as patient-reported outcomes (PROs), into 1 coherent dashboard that could be easily reviewed by physicians and patients as part of clinical workflows and clinically actionable. Here, we describe the HCD process resulting in the development of PD-BRIDGE according to proposed reporting guidelines for health research involving design [14]. We also report initial user experience.

## Methods

### Clinical and Research Setting

All study activities took place at the University of California, San Francisco (UCSF) Movement Disorders and Neuromodulation Center, a tertiary academic referral center with a busy clinical practice involving people with PD and other movement disorders. Our research team included clinicians with specialization in movement disorders (EB, IB, JO, and CT), clinicians with experience in digital health applications (RB, KR, and SS), a software engineer (ES), and a participant coordinator and data analyst (WR). These roles were chosen to guarantee familiarity with the challenges of clinical encounters with people with PD and the scope of how digital health solutions could address these challenges. PROs were completed remotely in the patients’ homes prior to each visit, and some clinical consultations involved telemedicine.

### Design Process

#### Phase I: Inspiration Phase

The first phase of HCD is focused on understanding the problem and empathizing with the users. PD-BRIDGE was adapted from BRIDGE, an established platform launched directly from the EHR to pull clinical data in real time to be actionable during the clinical encounter. For adaptability, BRIDGE was designed as a Substitutable Medical Applications and Reusable Technology on Fast Health Interoperability Resources (SMART on FHIR) [15,16] interface, which provides the technical capability for implementation in different EHR vendors and usage of different applications. BRIDGE converts multiple information streams (clinic-specific flow sheets, imaging data, patient questionnaires, and other EHR data elements) into visualization modules, or “widgets,” that can be customized accordingly to fit the needs of users with various clinical conditions, including multiple sclerosis [10] and other neurologic and neuropsychiatric diagnoses. Therefore, our first step was to identify the most useful modules to develop for PD-BRIDGE.

We began our inspiration phase with 2 focus groups, 1 with 14 movement disorders experts, as well as 5 individual discussions with people with PD. In this phase, we identified challenges associated with systematically collecting and visualizing data, understanding patient histories, and gathering and understanding patients’ daily patterns for medication adherence and side effects. The inspiration phase occurred over the course of 3 months.

#### Phase II: Ideation Phase

The ideation phase of HCD is focused on creating solutions for the problems defined in the inspiration phase. Through the focus groups, we generated mock-ups for preferred data visualization modules that could support the clinical information most often discussed and required for making decisions in clinical encounters with PD patients. Preliminary mock-up designs of these ideas were constructed and then presented to 5 physician/patient stakeholders for more feedback before implementation. Feedback was centered around summarizing complex histories, visualizing elements that could support medication management, and better visualization of longitudinal progression. The modules were then programmed and implemented on a live platform and made available to a select group of testers. The ideation phase occurred over the course of 6 months.

#### Phase III: Implementation Phase

The implementation phase is focused on building, testing, and iterating the solution. Once the PD-BRIDGE dashboard was built, user testing involved 2 stages. In the first stage, 2 movement disorders physicians—selected for their clinical volume and enthusiasm for digital technologies—agreed to use the platform for their upcoming regularly scheduled clinical encounters and to display it to their patients as appropriate. The clinicians then provided feedback regarding any potential software bugs, accuracy related to the medical chart, and requirement of further coding (eg, whether to code and display different formulations of a medication as 1 or 2 separate medications). Patients in these encounters were invited to fill out PROs before their scheduled visit and provide qualitative feedback regarding the usability and relevance of the tool after their clinical encounter. Further programmatic development then occurred to address qualitative feedback arising during this stage. This first stage took place over 3 months.

In the second stage, all 14 movement disorders physicians in our center were invited to use the platform in their upcoming regularly scheduled clinical encounters, with a goal of 30 total encounters. Each participating clinician signed an informed consent. Clinicians would identify patients having complex symptoms amenable to PD-BRIDGE and notify the research coordinator so they could be contacted in advance of the visit over telephone. After obtaining informed consent, the research coordinator would obtain demographic information, basic information about the disease state, and then send the PROs to the participants.

During the clinical encounter, clinicians launched the PD-BRIDGE platform; both sets of users were then invited to complete user experience surveys (see Multimedia Appendices 1-2). Survey questions were developed based on the Health Information Technology Usability Evaluation Model [17]. This model integrates multiple usability theories including the Technology Acceptance Model (TAM) [18] and evaluates subjective (eg, perceived usefulness, perceived ease of use) and objective (eg, efficiency, effectiveness) outcomes. Specifically, patient surveys focused on the usefulness of PD-BRIDGE, the quality of communication throughout the encounter, comfort with the implementation and perceived security of the platform, and overall satisfaction of the visit. Clinician surveys focused on completeness of the data available, usability of PD-BRIDGE, the ability of the platform to facilitate the encounter, and overall satisfaction with the visit. Both surveys asked for specific feedback about visualizations and data in PD-BRIDGE. All surveys were in English and were administered using the REDCap platform; REDCap was also used to store survey responses. This second stage of implementation occurred over the course of 6 months, leading to a total design process timeline of 18 months.

### Data Analysis

We constructed descriptive tables to summarize the demographic and clinical information about the participant cohort. We then summarized survey response data from clinicians and participant respondents. All descriptive analyses were performed using R (version 4.0.2; R Foundation for Statistical Computing). Tables were generated using the gtsummary package [19] and graphs were constructed using the ggplot2 package in R [20].

### Approvals and Consent

Evaluation of the platform and responses to the questionnaires were approved by the UCSF Institutional Review Board (IRB # 18-26148). Electronically signed informed consent was obtained from clinicians and patients prior to their participation.

## Results

### PD-Specific BRIDGE Modules Designed and Developed Through the HCD Process

**Figure 1:**
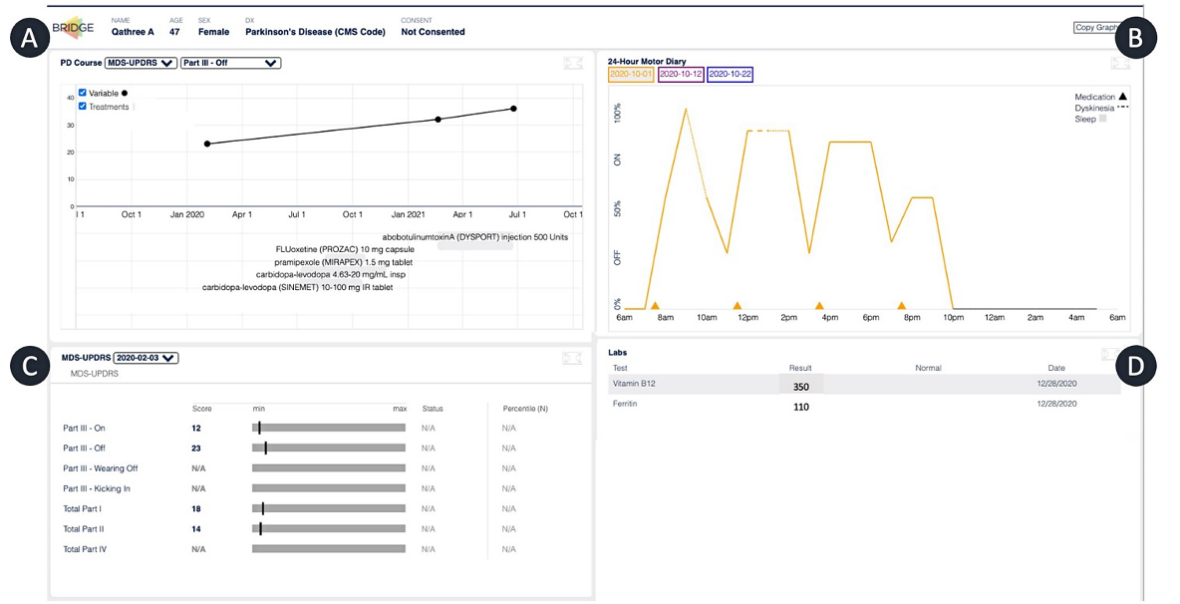
Mock-up of the Parkinson disease–specific BRIDGE platform with (A) longitudinal measurement of motor scales, (B) visualization of a patient-entered motor diary, (C) cross sectional clinical scores, and (D) prior diagnoses and laboratory studies relevant to Parkinson disease. HCC: hierarchical condition category.

Through stakeholder discussions, several modules were designed that would provide meaningful information during a clinical encounter with a person with PD (Figure 1).

### Adaptation of Existing Modules

To provide historical context to patients and clinicians, information about PD-related medication use and graphical display of the Movement Disorders Society Unified Parkinson’s Disease Rating Scale scores were determined to be highly relevant. These visualizations could be adapted from existing BRIDGE modules, with PD-specific data pulled from the EHR and displayed longitudinally (Figure 1A) as well as cross-sectionally in more detail (Figure 1B). Additionally, laboratory studies and comorbid conditions that are relevant to symptoms and medication management in PD were pulled from the EHR (Figure 1D).

### New Module

Stakeholders agreed that a module displaying changes in motor symptoms over the course of the day based on a prospectively collected diary would facilitate clinical decision-making around medication changes and represent an advancement over patient/caregiver recall alone. Having reviewed the existing literature and iOS/Android app stores for such a feature and finding none, we designed a specific module to visualize data from a motor diary prospectively collected as an electronic PRO over the course of 24 hours prior to a patient’s visit. Our survey was initially based on the original paper diary for PD designed by Hauser et al [21], asking participants to define whether they were in the medication “ON” or “OFF” state, or had dyskinesia involving abnormal involuntary movements that occur as a complication of medications for PD that were either troublesome or not troublesome. However, patients noted trouble with the binary choice between ON and OFF and requested more granularity for choosing to what extent their medications were working. Therefore, we adapted the diary to allow for a graded response (see Multimedia Appendix 3), adjusted the visualization to express medication effects over the course of the day from a scale of 0% to 100%, and overlaid them with indicators of the presence of dyskinesia and medication timing (Figure 1B). Thus, the ON time for a given participant could be interpreted as the area under the curve and used to justify and discuss changes in medication timing or dosage.

### Modules Prioritized for a Future Round of Development

Additional modules were deemed desirable by some participants but were postponed to a future round of development after the initial testing phase. These modules either had a lower overall priority according to stakeholders or required a greater technical “lift.” The themes of these proposed modules include understanding longitudinal changes in nonmotor symptoms of PD (including mood and cognition), visualizing scores of neuropsychiatric testing, integrating neuroimaging and clinical videos, and visualizing changes in deep brain stimulation settings. PD-BRIDGE can also display quantitative motor and nonmotor ratings in relation to averages across the clinic or compared to age-adjusted normative values. Clinicians felt that these features would not be appropriate because advanced patients could be discouraged by seeing their information in this context, scores could be subjective, and normative values were not well established for PD. Hence, we did not include these features in this iteration but asked participants for their opinions in this area.

### Implementation Phase: Patient Experience

In total, 34 patients consented to test the PD-BRIDGE platform in their clinical encounter and 32 filled out the motor diary. Of these, 21 completed the user experience survey after their visit (Table 1). Demographic and clinical characteristics of those who completed the survey were not significantly different from those who did not (data not shown). Overall, PD-BRIDGE was used in at least 17 clinical encounters; in 1 additional encounter, a clinician reported using PD-BRIDGE whereas a patient did not.

**Figure 2:**
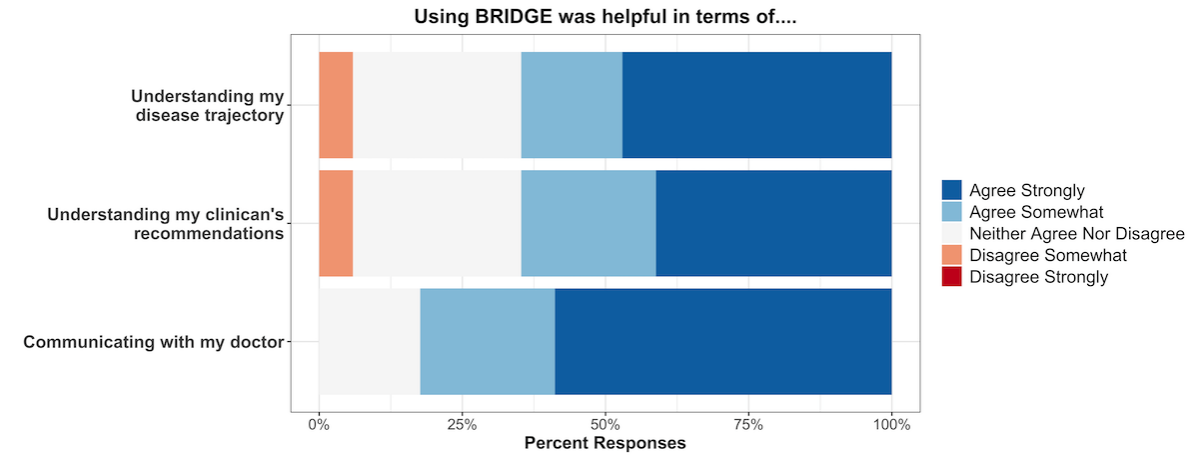
Responses from patients regarding the helpfulness of using PD-BRIDGE.

**Figure 3:**
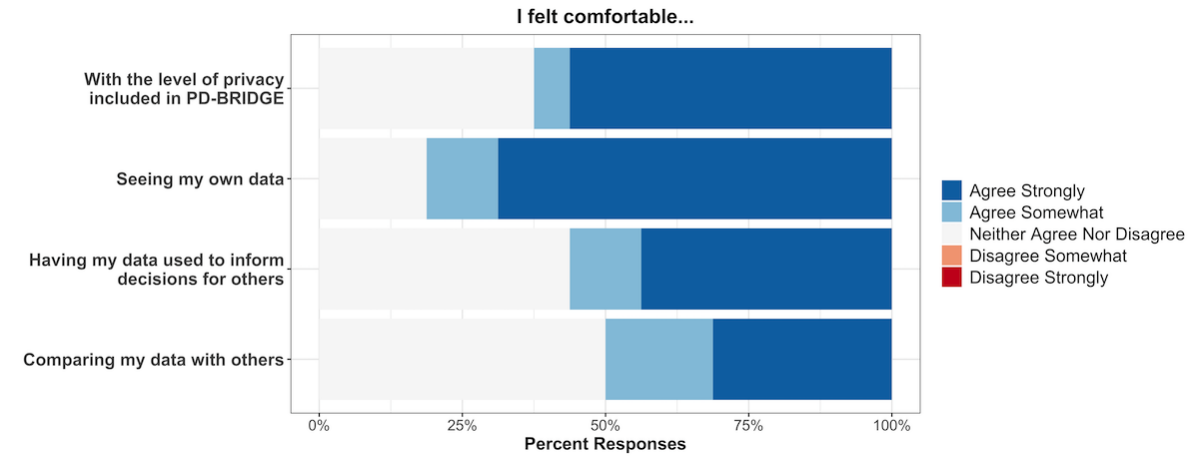
Responses from patients pertaining to their comfort with different aspects of PD-BRIDGE.

Of the 17 patients reporting that their physician used PD-BRIDGE during their clinical encounter, 14 (83%) felt that PD-BRIDGE facilitated communication with their clinician, 11 (65%) thought it helped them understand their disease trajectory, and 11 (65%) felt that it helped them understand their clinician’s recommendations (Figure 2). Participants felt comfortable visualizing their own data points, and though not currently a feature of the PD-specific BRIDGE, 9 (50%) stated that they felt comfortable comparing their data with others’ deidentified data, and 10 (56%) felt comfortable having their deidentified data used to inform decision-making for others. Importantly, no participants expressed concerns about the privacy of their data on the PD-BRIDGE platform (Figure 3).

**Table 1:** Characteristics of patient users of PD-BRIDGE (N=17).

Characteristic		Value
Age at visit in years, mean (SD)		66 (11)
Males, n (%)		11 (65)
Not Hispanic or Latino, n (%)		17 (100)
**Race, n (%)**		
	Other	1 (5.9)
	Unknown	1 (5.9)
	White	15 (88)
Disease duration in years, mean (SD)		6.8 (4.0)

Specific comments were solicited from patient participants about what did and did not work well about the PD-BRIDGE platform. With respect to ease of use, 3 participants remarked that filling in data was fast and easy, whereas 1 found filling out the motor diary challenging. With respect to the likeability and usefulness of the visualizations, 5 appreciated the visualizations. Qualitative comments included feelings that PD-BRIDGE provided “a clearer picture of how (medications were) working at different times throughout the day/night,” that the “graph was helpful in explaining…symptoms during the day,” and that it showed “visual progression.” One participant specifically noted that discussing the visualizations facilitated “adjustment of…medication as it relates to wearing-off.” Another patient remarked that the platform did not yet include enough data to be helpful.

### Implementation Phase: Neurologist Experience

In total, 11 movement disorders neurologists filled out a survey for a patient encounter, for a total of 16 encounters where PD-BRIDGE was used. With respect to data and visualizations, neurologists generally felt that they had the correct visual aids to explain their patients’ disease to them (agreeing for n=11, 69% of encounters) and disagreed that they had difficulty communicating with their patient about their disease course (n=14, 88%) or their recommended care (n=15, 93%). Neurologists felt that PD-BRIDGE helped them “get on the same page” as their patient 94% (n=15) of the time. Further, although neurologists felt that the data were up to date in 94% (n=15) of the encounters, they felt the data were not exhaustive in 62% (n=10) and that the EHR was more complete in 50% (n=8) of encounters. Still, during 44% (n=7) of the encounters, neurologists felt that the data in PD-BRIDGE were more complete than what could be retrieved through the EHR. In terms of improvements, 4 neurologists suggested that more data be imported into PD-BRIDGE to make it applicable to a wider range of patients, whereas others requested more features such as visualization of nonmotor symptom progression (1 neurologist) or uploading of clinical videos (1 neurologist).

**Figure 4:**
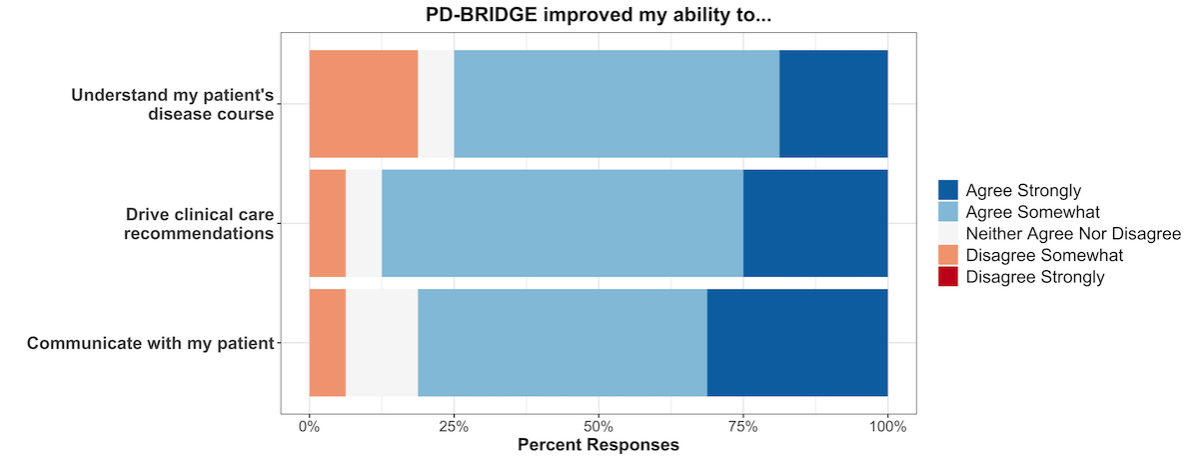
Responses from clinicians regarding clinical aspects of PD-BRIDGE.

With respect to perceived usefulness, neurologists felt that PD-BRIDGE helped with many clinical aspects of the office visit in the majority of the 16 encounters, including understanding their patients’ disease course (n=12, 75%), driving clinical care recommendations (n=14, 87%), and communicating with their patient (n=13, 81%), as shown in Figure 4. When asked about specific features that worked, they mentioned the benefit of “visualizing (that) fluctuations were greatly reduced,” “demonstrat(ing) the need for increasing medications,” and “hav(ing) a visual aid for patients and clinicians to reference and to guide discussion.” Neurologists appreciated seeing “the (clinical) trajectory longitudinally” and the “ability to incorporate the MDS-UPDRS.”

## Discussion

### Principal Results

Using iterative design techniques and engaging feedback from intended patient and clinician users, we developed a disease-specific platform designed to facilitate clinical encounters for the care of people with PD.

Although information overload occurs throughout different areas of medicine, PD is a condition that would particularly benefit from integrated and efficient delivery of clinical information. Symptoms in PD change frequently over the course of a typical day and vary from patient to patient, making modules such as the motor diary helpful to not only capture hour-by-hour fluctuations in patient experience but also visualize these reports efficiently. These symptoms will likely be captured by mobile or wearable devices in the near future, creating another information stream that needs to be effectively displayed in the EHR. Furthermore, PD management involves multiple types of medications and procedures (eg, deep brain stimulation and botulinum toxin), which can evolve and become increasingly complex over time, resulting in a large amount of information to review and consider at each clinic visit. Designing ways to rapidly ingest and use this data to inform clinical decisions and assist in counseling patients is imperative.

We used an HCD process to develop a SMART on FHIR application to approach this problem. We decided that the HCD process would most effectively solicit the varied perspectives involved in clinical care of people with PD with an instrument that could then be adopted quickly into clinical workflows. We also felt that the SMART on FHIR platform and modular approach of BRIDGE allowed for the greatest adaptability to various needs and would guarantee eventual transferability to other contexts. The HCD process was indeed effective in engaging clinicians and patients in all stages of design and allowed us to incorporate clinical workflows into platform development. However, a major challenge with this process was how to prioritize various requests and perspectives; even within the same subspecialty, clinicians have various workflows and find different types of information useful, such that selecting the most important and most widely applicable visualizations was challenging. Future iterations of PD-BRIDGE will include a wider array of modules to meet these requests, and the modular approach will allow for further personalization. We also noted that stakeholder enthusiasm for aspects of PD-BRIDGE, expressed in focus groups, did not always translate to engagement, and incorporating pilot testing and implementation into the design phase was an essential aspect of HCD to identify the barriers to adoption of the platform.

PD-BRIDGE facilitates clinical encounters by integrating information from disparate sources (eg, home diaries, elements in the EHR) into easily visualized displays. The modular design of the platform allows for easy adaptation to various subspecialties. Our PD stakeholders were enthusiastic about this platform and readily identified several key features that would increase efficiency in clinical encounters. Some were prioritized for the current version, and others will be integrated into future versions. However, the perceived benefit of a digital health product is not sufficient for its adoption, and clinical workflows can be especially challenging processes to change. The likelihood that a digital health instrument such as PD-BRIDGE will be used can be evaluated using a proposed structured framework that considers technical validation, clinical validation, usability, and cost [22].

From a technical perspective, the information that PD-BRIDGE represented was accurate when compared to the current gold standard, namely the EHR. The main feedback from neurologists for the PD-BRIDGE platform was that data were not as complete as that in the EHR. Although the EHR has a wealth of clinically relevant information, much of it is buried in free text and is challenging to extract in an automated and a reliable fashion. To address these issues, we developed an EHR flowsheet in parallel where specific clinically relevant information is collected in a manner accessible to PD-BRIDGE for integration in future versions. Notably, our institution’s EHR (EPIC) makes flow sheets available to other institutions’ developers through EPIC App Orchard, meaning that they can be easily downloaded and used in other subspecialty clinics with the same EHR. Another important technical feature of the EHR is privacy, which BRIDGE maintains by launching directly from within the EHR firewall. Reassuringly, patients did not express any concerns with PD-BRIDGE threatening privacy of their data.

From a clinical perspective, the major goal of PD-BRIDGE was to facilitate discussions about symptom management, which can be complex in PD. In our discussions, movement disorders neurologists indicated that their patient counseling usually relies on purely verbal conversations with no visual aid, though reading material may be provided to a patient afterward. Some neurologists did use illustrations to convey their messages, but these were usually not patient-specific. PD-BRIDGE transformed patient-specific data into visualizations that were rated as clinically useful by patients and providers; having a visual aid helped translate the patients’ verbal description of their symptoms and improved their understanding of the purpose of medication changes. Some patients also noted that PD-BRIDGE facilitated longitudinal understanding of their condition, which is a challenging disease characteristic to grasp from the EHR. In initial focus groups, neurologists had expressed a concern about patients visualizing how their data compared to others or allowing their data to be even seen in the aggregate form. Although our version of PD-BRIDGE did not include these features, it was reassuring that the patients we surveyed indicated no specific concern around these issues, and such features may be worthwhile to include in the future.

PD-BRIDGE also demonstrated sufficient usability, though we identified areas for improvement. The majority of PD-BRIDGE data are pulled automatically from the medical record, and this therefore places minimal burden on the clinician users, who can access all these data with a click at the POC. However, data must be entered discretely to be used in visualizations, and as PD-BRIDGE begins to incorporate more data streams, clinicians may need to change how they enter clinical data. PD-BRIDGE also relies on patient-entered PROs, including the 24-hour motor diary visualizations, which did require time from patients. Some patients appreciated the opportunity to list their symptoms, and some found it burdensome and error-prone. Therefore, these aspects of PD-BRIDGE may be more amenable to some clinicians and patients and not to others, and understanding these opinions can inform future implementation efforts. Future advances, such as a voice input option in lieu of keyboarding, may improve accessibility for motor-impaired patients.

### Limitations

Despite this encouraging preliminary feedback, the current study’s limitations require that further testing be conducted. We were only able to survey a small number of patients and neurologists and may not have captured the full range of feedback on the instrument. The results of the survey may also have suffered from selection bias; although we included the majority of movement disorders neurologists in our division, the groups of patients willing to test PD-BRIDGE were possibly already enthusiastic about this type of technology, even if we did not observe significant differences between participants and nonparticipants. Future studies can evaluate how PD-BRIDGE improves clinician efficiency, such as by reducing clicks in the EHR, or improves health outcomes, such as by emphasizing important topics from the patient perspective or reminding physicians of quality metrics.

### Conclusions

Overall, this study shows the usefulness of adapting a platform that exists within the EHR to subspecialty-specific use. Future versions of PD-BRIDGE will integrate more information streams, such as images, clinical videos, PROs that capture more aspects of PD, and patient devices. As the complexity and breadth of clinical care in PD increases, such a platform will be essential to translate the wealth of information into actionable clinical care.

## Appendix

Multimedia Appendix 1 Patient postvisit questionnaire.

Multimedia Appendix 2 Clinician postvisit questionnaire.

Multimedia Appendix 3 Parkinson disease motor diary example.

## Abbreviations

EHR: electronic health record
HCD: human-centered design
PD: Parkinson disease
POC: point of care
PRO: patient-reported outcome
SMART on FHIR: Substitutable Medical Applications and Reusable Technology on Fast Health Interoperability Resources
