# Supplementary material for: Enhancing Clinical Information Display to Improve Patient Encounters: Human-Centered Design and Evaluation of the Parkinson Disease-BRIDGE Platform
Source: JMIR Hum Factors. 2022 May 6;9(2):e33967. doi: 10.2196/33967 (PMC9123539; doi:10.2196/33967)
Supplement: Multimedia Appendix 1 [file humanfactors_v9i2e33967_app1.pdf]

This is a Multimedia Appendix to a full manuscript published in the J Med Internet Res. For full copyright and citation information see http://dx.doi.org/10.2196/jmir.33967

Record ID ______________________________

Today's Date ______________________________

Full Name ______________________________

Clinician ______________________________

We would like to ask you some questions about your visit today. Please note that these answers will not be shared with your provider except in anonymous, aggregated form.

Thinking about today's visit with your movement disorders doctor, how satisfactory was your doctor at each of the following?

	Very Good	Good	Neither Good Nor Poor	Poor	Very Poor	Doesn't Apply
Spent sufficient time with you	○	○	○	○	○	○
Asking about your symptoms	○	○	○	○	○	○
Listening to you	○	○	○	○	○	○
Explaining tests and treatments	○	○	○	○	○	○
Involving you in decisions about your care	○	○	○	○	○	○
Treating you with care and concern	○	○	○	○	○	○
Taking your problems seriously	○	○	○	○	○	○

Overall, do you feel satisfied with today's visit?
- ○ Yes
- ○ No

BRIDGE is a web-based instrument / clinical dashboard that your doctor may have used in your visit today to discuss your symptoms.

Did your doctor use the BRIDGE tool in your visit today?
- ○ Yes
- ○ No

We would appreciate your feedback about BRIDGE.

Using BRIDGE was helpful for my visit, in terms of:

	Agree Strongly	Agree Somewhat	Neither Agree Nor Disagree	Disagree Somewhat	Disagree Strongly
Communicating with my doctor	○	○	○	○	○
Understanding my disease trajectory	○	○	○	○	○
Understanding my clinician's recommendations	○	○	○	○	○

Now, a few questions about the data shown in BRIDGE:

I felt comfortable...

	Agree Strongly	Agree Somewhat	Neither Agree Nor Disagree	Disagree Somewhat	Disagree Strongly
Seeing my own data points	○	○	○	○	○
Comparing my data with other patient's data	○	○	○	○	○
Having my data used to inform decision making for other patients	○	○	○	○	○
With the level of privacy of my data included in the BRIDGE tool	○	○	○	○	○

Finally, please let us know:

What worked well for you? ______________________________

What did NOT work well for you? ______________________________

Would you want the opportunity to consult directly with a member of the BRIDGE research team? ○ Yes ○ No
