# Supplementary material for: Enhancing Clinical Information Display to Improve Patient Encounters: Human-Centered Design and Evaluation of the Parkinson Disease-BRIDGE Platform
Source: JMIR Hum Factors. 2022 May 6;9(2):e33967. doi: 10.2196/33967 (PMC9123539; doi:10.2196/33967)
Supplement: Multimedia Appendix 2 [file humanfactors_v9i2e33967_app2.pdf]

This is a Multimedia Appendix to a full manuscript published in the J Med Internet Res. For full copyright and citation information see http://dx.doi.org/10.2196/jmir.33967

Record ID	__________________________
Today's Date	__________________________
Clinician Name	__________________________
Patient Name	__________________________
Patient Code	__________________________

First, a few questions about today's visit.

To what extent do you agree or disagree with the following statements?

	Agree Strongly	Agree Somewhat	Neither Agree Nor Disagree	Disagree Somewhat	Disagree Strongly
I am globally satisfied with my visit with the patient	◯	◯	◯	◯	◯
I was able to tackle the issues important to my patient	◯	◯	◯	◯	◯
I had the right visual aides to explain my patient's disease to them	◯	◯	◯	◯	◯
I spent a long time looking through my patient's record in Apex	◯	◯	◯	◯	◯
In communicating with my patient, it was appropriate to see their disease course in the context of a wider cohort	◯	◯	◯	◯	◯
I had some difficulty communicating with my patient about their disease course	◯	◯	◯	◯	◯
I had some difficulty communicating with my patient about my care recommendations	◯	◯	◯	◯	◯
I don't have access to all the data relevant to caring for my patient	◯	◯	◯	◯	◯

Was BRIDGE used during this visit? ◯ Yes ◯ No

BRIDGE helped me "get on the same page" as my patient.

◯ Agree Strongly ◯ Agree Somewhat
◯ Neither Agree or Disagree
◯ Disagree Somewhat ◯ Disagree Strongly

To what extent do you agree or disagree with the following statements?

The data retrieved by BRIDGE about my patient...

	Agree Strongly	Agree Somewhat	Neither Agree Nor Disagree	Disagree Somewhat	Disagree Strongly
was exhaustive	◯	◯	◯	◯	◯
was up to date	◯	◯	◯	◯	◯
was more complete than the data I can retrieve through the EHR	◯	◯	◯	◯	◯

To what extent do you agree or disagree with the following statements?

BRIDGE as a whole improved my ability to...

	Agree Strongly	Agree Somewhat	Neither Agree Nor Disagree	Disagree Somewhat	Disagree Strongly
understand my patient's disease course	◯	◯	◯	◯	◯
drive clinical care recommendations	◯	◯	◯	◯	◯
communicate with my patient	◯	◯	◯	◯	◯

What worked well?

____

What could be improved?

____
