# Supplementary material for: Enhancing Clinical Information Display to Improve Patient Encounters: Human-Centered Design and Evaluation of the Parkinson Disease-BRIDGE Platform
Source: JMIR Hum Factors. 2022 May 6;9(2):e33967. doi: 10.2196/33967 (PMC9123539; doi:10.2196/33967)
Supplement: Multimedia Appendix 3 [file humanfactors_v9i2e33967_app3.pdf]

This is a Multimedia Appendix to a full manuscript published in the J Med Internet Res. For full copyright and citation information see http://dx.doi.org/10.2196/jmir.33967

2:00 PM:

Please answer these questions based on how you felt most of the time over the last hour ***(between 1:01 PM and 2:00 PM).***

Were you asleep for most of the last hour (between 1:01 PM and 2:00 PM)?

* must provide value

Yes

No

reset

Please move the slider to indicate how well your medications were working ***most of the time between 1:01 PM and 2:00 PM***. Completely OFF, or all the way to the left, indicates that medications have not been working at all. Completely ON, or all the way to the right, indicates that medications had **the best effect that they can have in your case**.

* must provide value

Completely OFF | Somewhat ON | Completely ON

30

reset

Please move the slider to indicate how severe dyskinesia were, if present, ***most of the time over the last hour (between 1:01 PM and 2:00 PM).*** All the way to the right, or 0, indicates no dyskinesia. Halfway indicates dyskinesia were present but not troublesome, and all the way to the left indicates dyskinesia were present and very troublesome.

* must provide value

No dyskinesia | Dyskinesia present but not troublesome | Dyskinesia present and very troublesome

0

reset
